# Iodine as a Topical Application

**Published:** 1855-01

**Authors:** 


IODINE AS A TOPICAL APPLICATION.—The topical application of this remedy is becoming very general in London. The following is the formula: ℞ Iodinii ʒj., sp. vini rect. ℥.; ft. solutio. It should be applied in glandular affections beyond and around the enlarged parts, so that the absorbed fluid may be carried through the gland by the lymphatic vessels. It is used topically, 1st, in pleuritic and neuralgic stitches; 2nd, to the throat in cases of aphonia or hoarseness; 3rd, to the mucous lining of the throat itself in cases of congestion and of enlargement of the tonsils; 4th, around the external parts of the eye, in cases of strumous inflammation; 5th, in all forms of periostitis, whether syphilitic or strumous; 6th, in glandular affections as above mentioned; 7th, as injections into crysts and cavities of abscesses, provoking adhesive, but not suppurative inflammation, as in hydrocele.—*Med. Times and Gazette.*

